# Study of Correlation between Fetal Bowel Dilation and Congenital Gastrointestinal Malformation

**DOI:** 10.3390/children11060670

**Published:** 2024-05-31

**Authors:** Yi Jiang, Weipeng Wang, Weihua Pan, Wenjie Wu, Dan Zhu, Jun Wang

**Affiliations:** 1 Department of Pediatric Surgery, Xinhua Hospital Affiliated to Shanghai Jiao Tong University School of Medicine, No. 1665, Kongjiang Rd., Shanghai 200092, China; grace@sjtu.edu.cn (Y.J.); wangweipeng@xinhuamed.com.cn (W.W.); panweihua@xinhuamed.com.cn (W.P.); wuwenjie@xinhuamed.com.cn (W.W.); 2 Department of Pediatric Surgery, Hangzhou Children’s Hospital, Hangzhou 310000, China; 3 Department of Cardiovascular Surgery, Shanghai Chest Hospital, Shanghai Jiao Tong University, No. 241, West Huaihai Rd., Shanghai 200030, China; zhudanmd@sjtu.edu.cn

**Keywords:** fetus, bowel dilation, congenital gastrointestinal malformation

## Abstract

Background: Ultrasound serves as a valuable tool for the early detection of fetal bowel dilatation, yet the correlation between fetal bowel dilatation and gastrointestinal malformations remains to be further investigated. This study aims to explore the relationship by conducting a follow-up and analysis of fetuses with bowel dilation. Methods: A retrospective analysis was conducted on 113 fetuses with bowel dilatation at our center from July 2014 to December 2019. The location and degree of bowel dilatation were analyzed. ROC curves were constructed based on the diameter of the bowel and its ratio to fetal gestational age. Results: In total, 40 of 41 cases (97.6%) with upper gastrointestinal dilatation (double-bubble sign) and 46 of 72 cases (63.9%) with lower gastrointestinal dilatation were diagnosed with gastrointestinal malformations postnatally. The AUC of the dilatation diameter was 0.854 with a cutoff value of 18.05 mm in patients with lower gastrointestinal dilatation. The ratio of the diameter to gestational age (D/GA) showed a higher AUC of 0.906 with a cutoff value of 0.4931. Conclusions: The presence of the double-bubble sign in fetuses indicates a close association with duodenal obstruction. The risk of gastrointestinal malformations increases when the bowel diameter exceeds 18.05 mm, particularly when the D/GA surpasses 0.4931.

## 1. Introduction

In recent years, advancements in imaging modalities and the widespread use of prenatal examinations have enabled the detection of congenital gastrointestinal malformations, including intestinal atresia, intestinal stricture, Hirschsprung’s disease, and fetal fecal peritonitis, through fetal magnetic resonance imaging (MRI) and ultrasound before birth [1,2]. Many studies reported children with congenital gastrointestinal malformations often exhibit abnormal abdominal signs during the fetal period, such as bowel dilatation, hyperechogenic bowel, ascites, intra-abdominal masses, and polyhydramnios, but the accuracy of prenatal diagnosis based on these indicators remains limited [1,3,4]. Catania VD et al. [5] found that hyperechogenic bowel disappeared spontaneously during the fetal period in 1% of fetuses with hyperechogenic bowel. The incidence of postnatal gastrointestinal malformations was 36% in fetuses with hyperechogenic bowel found after 25 weeks of gestation. Muru A et al. found that fetuses with ascites before 24 weeks had a 10% risk of gastrointestinal malformations, which increased to 47% when ascites were detected after 24 weeks [6]. Gokcen et al. reported that the accuracy of prenatal diagnosis in their center was 52% for duodenal obstruction, 40% for jejunoileal obstruction, and only 20% for colonic obstruction [7]. F. He et al. indicated that the coexistence of ascites, bowel dilatation, and intra-abdominal cysts on fetal MRI suggested fetal fecal peritonitis [8]. These findings demonstrate that prenatal ultrasound and MRI, when used to identify abnormal signs, can serve as valuable tools in guiding the diagnosis and management of neonatal gastrointestinal malformations.

Prenatal ultrasound and MRI assessments are often influenced by maternal amniotic fluid volume, fetal position, and other factors. Additionally, some gastrointestinal signs will change with gestational age. Selecting digestive tract signs that can be dynamically monitored for regular follow-up of the fetus to assess the risk of postnatal gastrointestinal malformations has practical clinical application value for prenatal counseling and early postnatal treatment. Among them, dilated bowel diameter serves as the most direct indicator of fetal intestinal changes and is used for fetal evaluation in some maternal–fetal medical centers. Clinically, bowel diameter > 10 mm is often used as the cutoff for fetal bowel dilatation; however, the correlation between the degree of dilatation and gastrointestinal malformations remains to be explored. Lap Chiara et al. concluded that the average diameter of the fetal small intestine at 40 weeks of gestation was 5.1 mm based on ultrasound examination [9]. Lakhoo K et al. pointed intestinal obstruction should be considered when the diameter of the fetal bowel exceeds 7 mm and the length of dilated bowel exceeds 15 mm [10]. Suting Xu et al. proposed that judgments of bowel dilation should be made based on gestational age [11]. He pointed out that a diameter of the small intestine > 7 mm in any stage of gestation or a diameter of the colon > 18 mm in the third trimester can be considered as abnormal, suggesting gastrointestinal malformations with the fetus [11]. Therefore, we conducted this research to determine the relationship between the degree of bowel dilatation and gastrointestinal malformations.

## 2. Materials and Methods

We conducted a retrospective observational study of cases of fetal intestinal dilation (bowel diameter > 10 mm) followed at a tertiary center between July 2014 and December 2019. The basic information, including pregnancy information, fetal genetic testing results, imaging diagnosis, and other relevant information of the fetus and newborns was analyzed. Based on the prenatal ultrasound report, an amniotic fluid pocket depth ≥ 8 cm before 28 weeks of gestation or an amniotic fluid index ≥ 25 cm after 28 weeks of gestation was defined as polyhydramnios [12]. All children with prenatal intestinal dilation are required to have regular follow-up visits at our outpatient clinic after discharge. Telephone calls and online questionnaires were used to inquire about the follow-up results of these children. Various parameters during the obstetrical examination were recorded, including gestational age when bowel dilation, diameter and location of the dilated bowel, presence of other abnormal signs (such as hyperechogenic bowel, ascites, and intra-abdominal mass), and biometric indicators (biparietal diameter, femoral length, and abdominal circumference) were first detected. Postnatal manifestations (abdominal signs, meconium discharge, vomiting and other gastrointestinal symptoms), imaging examinations (abdominal X-ray, gastrointestinal imaging, abdominal MRI, etc.), and treatments were reviewed. The correlation between the degree of fetal gastrointestinal dilatation and malformation was investigated. For each ratio of the dilated bowel diameter to fetal gestational age (GA), biparietal diameter (BPD), femur length (FL), and abdominal girth (AG), the ROC curves were constructed correspondingly, and hence, the area under the curve (AUC), sensitivity, and specificity were derived.

The data were statistically analyzed with Microsoft Office-Excel Version 2016 and SPSS Version 23.0. Measurement data are expressed as the mean ± SD. The Kolmogorov–Smirnov test was used to test for data normality. The chi-square test, independent sample *t*-test, and Mann–Whitney U test were used for comparisons between the two groups. ANOVA was used for comparisons among multiple groups. *p* < 0.05 indicated a statistically significant difference.

## 3. Results

A total of 121 pregnant women were found to have a fetus with bowel dilatation and followed up. Five cases were twin pregnancy, and bowel dilatation was observed in only one of the twins in all these five cases. Five cases were in-vitro fertilization and one hundred sixteen were natural conception. In these cases, three chose to terminate the pregnancy due to fetal gene abnormalities, four cases underwent intrauterine death (one case of threatened preterm delivery, three cases of polyhydramnios and fetal embarrassment), and another one case accepted surgical treatment promptly after birth due to gastroschisis. Therefore, a total of 113 cases of fetal bowel dilatation were investigated in this study.

The average GA at first diagnosis of bowel dilation was 29.5 ± 5.5 weeks (range, 19^+5^–40^+6^). After birth, 86 neonates (*n* = 39 girls) were confirmed with intestinal atresia, intestinal stricture, annular pancreas, and other gastrointestinal malformations by physical examination, gastrointestinal imaging, and surgical exploration (Table 1). Twenty-seven patients were fed well after birth without manifestations of intestinal obstruction and showed normal growth during a follow-up from 6 months to 1 year. The overall incidence of congenital gastrointestinal malformations was 76.1% (86/113). Forty-six neonates (40.7%) had polyhydramnios during the fetal period, with an incidence of gastrointestinal malformations of 84.8% (39/46). Sixty-seven neonates (59.3%) had normal amniotic fluid volume during the fetal period, with an incidence of gastrointestinal malformations of 70.1% (47/67). There was no significant difference between the two circumstances (*p* = 0.073).

The cases were divided into two groups based on the site of fetal bowel dilatation on imaging examination: Group A comprised 41 cases, exhibiting dilation in the upper gastrointestinal tract (the segment proximal to the ligament of Treitz), while Group B encompassed 72 cases, demonstrating dilation in the lower digestive tract (the segment distal to the duodenum). The incidence of gastrointestinal malformation was higher in group A than in group B (97.6% vs. 63.9%, *p* < 0.001). There was no significant difference in the proportion of polyhydramnios between two groups.

### 3.1. Upper Gastrointestinal Dilatation and Duodenal Obstruction

**Figure 1:**
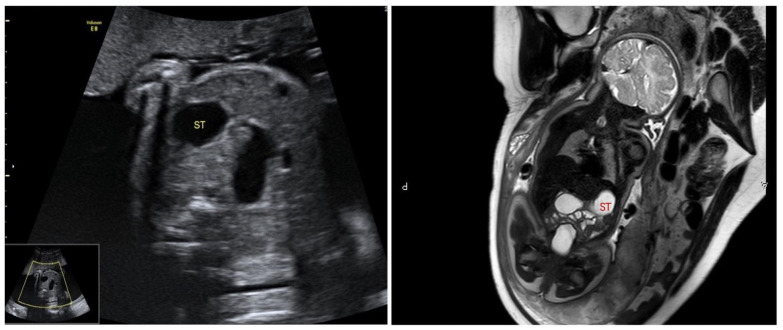
“Double-bubble” sign in ultrasound and MRI examination.

Based on the fetal ultrasound and MRI results, duodenal dilation was observed in 41 cases. In these cases, the dilated duodenum was connected to the gastric bubble, showing a typical sign like a double bubble (Figure 1). One hundred thirty-two ultrasound examinations were performed prenatally in these cases. GA at first time of diagnosis was 26.1 ± 4.5 weeks of gestation (range, 21^+6^–38^+1^ w). All these fetuses underwent an MRI examination prenatally and were confirmed with double-bubble sign. Subsequently, ultrasound was used to monitor the fetal development and changes in bowel dilatation. Each case received 3 ± 2 ultrasound examinations on average (1–9 examinations, mean = 3), with a continuous existence of double-bubble sign. Twenty-one cases were complicated with polyhydramnios. Forty newborns were confirmed to have congenital gastrointestinal malformations by surgical exploration in our department after birth, including twenty-one cases of annular pancreas (involving one case complicated with intestinal malrotation), sixteen cases of duodenal atresia (involving two cases complicated with intestinal malrotation), and three cases of atresia of the initial segment of the jejunum. Only one patient had no manifestations of intestinal obstruction after birth and was followed up for 1 year after discharge, with good growth and development. The accuracy of diagnosis of congenital duodenal obstruction using the fetal double-bubble sign was 97.6%.

The double-bubble sign was observed in 29 pregnant women in the second trimester, among which 14 cases (45.2%) were associated with polyhydramnios. The incidence of neonatal upper gastrointestinal malformations was 100% in these 29 cases. An annular pancreas was observed in 17 patients (58.6%), duodenal atresia was observed in 10 patients (34.5%), and atresia in the initial segment of the jejunum was observed in 2 patients (6.9%). In the other 12 cases, the double-bubble sign was first observed in the third trimester, involving 7 cases combined with polyhydramnios. In one case, the double-bubble sign was first observed at 38 + 1 weeks of gestation with normal amniotic fluid volume, and no manifestation of gastrointestinal abnormalities was observed after birth. Eleven of these twelve neonates were confirmed with upper gastrointestinal malformations by gastrointestinal imaging, with an incidence of 91.7%, incorporating six cases (54.5%) of duodenal atresia, four cases (36.4%) of annular pancreas, and one case (9.1%) of atresia in the initial part of the jejunum. The proportion of combinations with polyhydramnios and the incidence of gastrointestinal malformations were slightly higher in those diagnosed during the third trimester than those during the second trimester, but the differences were not significant (Table 2).

Among the 21 cases with polyhydramnios, GA at initial diagnosis of the double-bubble sign was 26.7 ± 5.0 weeks of gestation. Among the 20 cases with normal amniotic fluid volume, the GA at initial diagnosis of the double-bubble sign was 25.7 ± 4.1 weeks of gestation. No significant correlation was found in the above cases (*p* = 0.497).

### 3.2. Lower Digestive Tract Dilatation and Gastrointestinal Malformations

**Figure 2:**
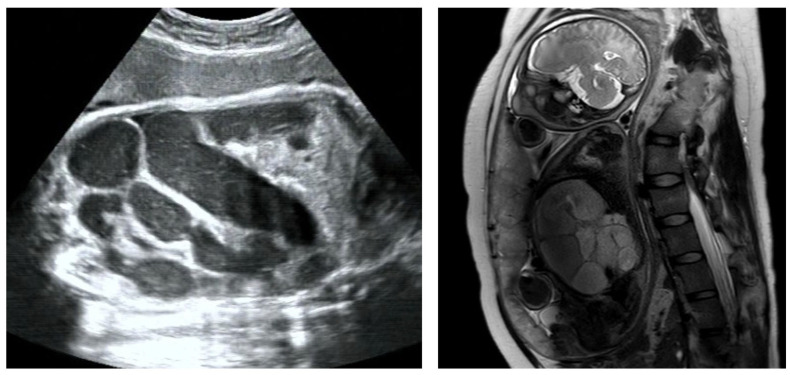
Bowel dilation in ultrasound and MRI examination.

Based on fetal ultrasound and MRI assessments, lower digestive tract dilatation was observed in 72 fetuses. In these cases, dilated bowel appeared separately, with no connection with gastric bubble, indicating that the dilatated segment was in the jejunoileum or colon (Figure 2). The average GA at first time of diagnosis was 31.4 ± 5.1 weeks of gestation (range, 19^+5^–40^+6^ weeks).

**Figure 3:**
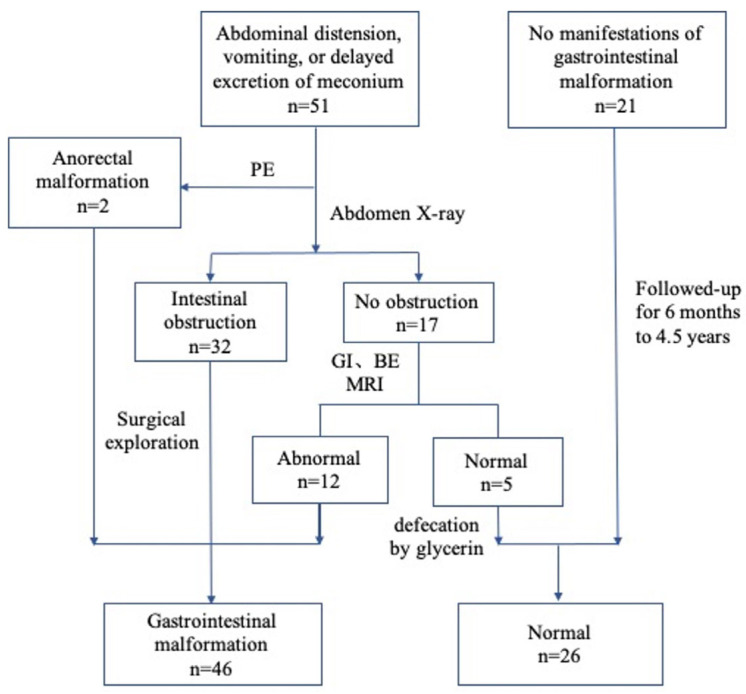
Gastrointestinal malformation in neonates with lower-bowel dilation in fetal period.

Among them, 21 (29.2%) neonates proved to be normal with no manifestations of abdominal distension, vomiting, or delayed excretion of meconium after birth. Fifty-one (70.8%) neonates underwent an abdominal X-ray examination after birth due to abdominal distension, vomiting, or delayed excretion of meconium. Among the 51 patients, 5 (9.8%) infants showed good tolerance to feeding after defecation assisted by glycerin, and 46 (90.2%) infants were identified with gastrointestinal malformations. The incidence of gastrointestinal malformations in this group was 63.9% (46/72) (Figure 3). Among the 46 infants, 32 (69.6%) were found to have intestinal obstruction using abdominal X-ray and surgical exploration; 2 (4.3%) neonates were found to have rectal stricture and cloacal malformation by physical examination; 12 (26.1%) neonates were found to have jejunoileal stricture/atresia or mesenteric cyst by gastrointestinal imaging (GI), barium enema (BE), abdominal MRI, and other subsequent examinations. The most common types of gastrointestinal malformations were intestinal atresia (76.1%), followed by intestinal stricture (6.5%) and mesenteric cyst (6.5%).

The neonates were arranged into two groups based on gastrointestinal malformation, with 46 cases in the gastrointestinal malformation group and 26 cases in the normal group. The average GA at first diagnosis with bowel dilatation in gastrointestinal malformation group was significantly earlier than that in normal group (29.8 ± 4.9 weeks vs. 34.3 ± 4.0 weeks, *p* < 0.001).

Among the 58 cases whose ultrasound and MRI examination both indicated bowel dilatation, 43 neonates were diagnosed with gastrointestinal malformation, whereas 3 of 14 cases with only the MRI indicator were diagnosed with gastrointestinal malformation. For ultrasound, the prenatal accuracy of diagnosis rate for gastrointestinal malformations was 74.1% (43/58), and for MRI, the accuracy rate was 63.9% (46/72). According to the location of dilated bowel on the MRI image, the cases were divided into the jejunoileum group (*n* = 43), colorectum group (*n* = 26) and diffuse dilatation group (*n* = 3). All the neonates in the diffuse dilatation group were confirmed with gastrointestinal malformation by surgery. Thirty-three of the forty-three neonates (76.7%) in the jejunoileal dilatation group had gastrointestinal malformation, while ten of the twenty-six neonates (38.5%) in the colorectal dilatation group had gastrointestinal malformation, demonstrating a high accuracy of diagnosis for jejunoileal malformation by fetal MRI.

**Figure 4:**
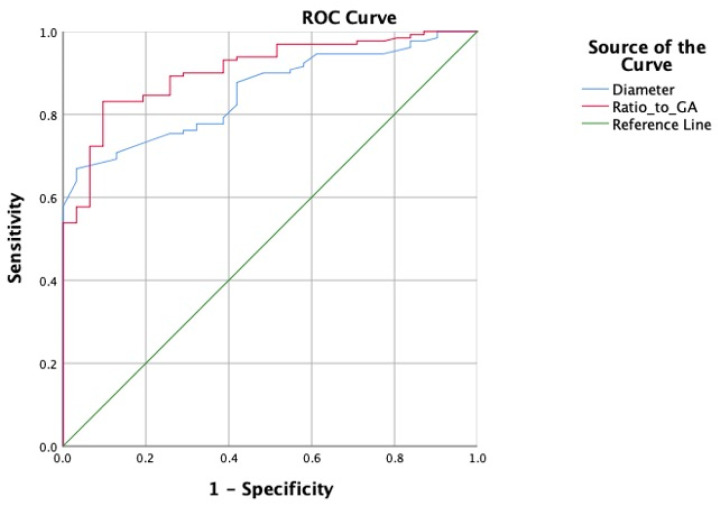
ROC curve of diameter of dilated bowel and ratio to gestational age (D/GA).

For bowel dilatation identified by ultrasound, a total of 170 prenatal ultrasound examinations were performed for 58 cases. The diameter of the dilated bowel diameter ranged from 11 to 57 mm, with an average of 21.2 ± 8.0 mm. The average GA at initial diagnosis was 30.8 ± 4.6 weeks of gestation (9^+5^–38^+3^ weeks). Thereinto, bowel dilatation was transient during fetal period in nine cases, involving two cases confirmed as gastrointestinal malformations after birth. In the rest of the 49 cases, bowel dilation persistently existed during pregnancy, and 41 proved to be with gastrointestinal malformation. To investigate the correlation between the degree of fetal gastrointestinal dilatation and malformation, we calculated the ratio of bowel diameter to gestational age (D/GA) and used ROC curves to analyze the predictive value of the diameter and the ratio (Figure 4). The AUC for diameter was 0.854 (95%CI: 0.795–0.913, *p* < 0.001), with a cutoff value of 18.05 mm, sensitivity of 0.669, and specificity of 0.968. The AUC for D/GA was 0.906 (95%CI: 0.856–0.956, *p* < 0.001), with a cutoff value of 0.4931, sensitivity of 0.831, and specificity of 0.903. That is, the ratio of D/GA showed better accuracy in predicting the risk of gastrointestinal malformations after birth.

**Figure 5:**
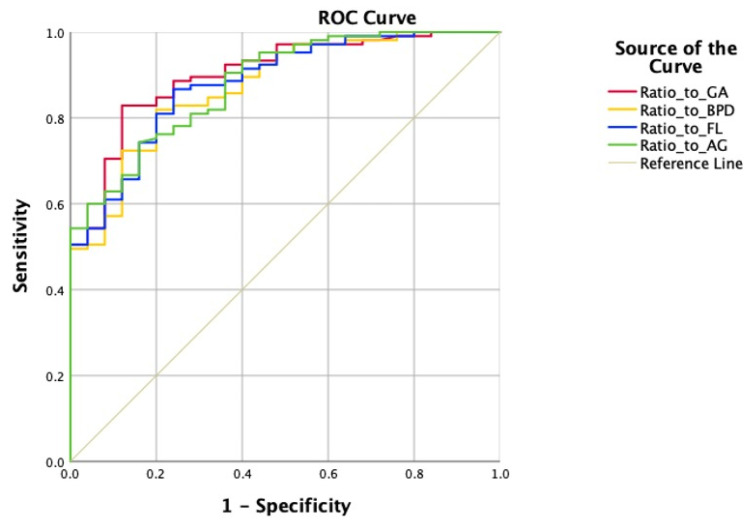
ROC curve of diameter of dilated bowel and ratio to GA, BPD, FL, AG.

Other than GA, biparietal diameter (BPD), femur length (FL), and abdominal girth (AG) are also important parameters to assess fetal development. We calculated each ratio of dilated bowel diameter to gestational age (D/GA), biparietal diameter (D/BPD), femur length (D/FL), and abdominal girth (D/AG). The ROC curves were constructed correspondingly, and hence, the area under the curve (AUC), sensitivity, and specificity were derived (Figure 5). We found that the AUC for D/BPD was 0.873 (95%CI: 0.803–0.943, *p* < 0.001), with a cutoff value of 0.1842, sensitivity of 0.819, and specificity of 0.800. The AUC for D/FL was 0.881 (95%CI: 0.813–0.948, *p* < 0.001), with a cutoff value of 0.2403, sensitivity of 0.867, and specificity of 0.760. The AUC for D/AG was 0.881 (95%CI: 0.815–0.947, *p* < 0.001), with a cutoff value of 0.0532, sensitivity of 0.743, and specificity of 0.840.

## 4. Discussion

In recent years, advancements in fetal ultrasound and MRI techniques have resulted in a gradually increasing detection rate of abdominal gastrointestinal signs such as bowel dilatation. Therefore, the diameter of the dilated bowel, as a direct indicator to assess the severity of dilation, has been used for prenatal diagnosis in some centers. Chiara et al. found that the average diameter of the small intestine was 2.4 mm and the average diameter of the colon was 3.9 mm at 24 weeks of gestation [9]. By 40 weeks of gestation, the average diameter of the small intestine increased to 5.1 mm, and that of the colon was up to 14.5 mm. For the fetal colon, the diameter was approximately 3 mm in the second trimester and then increased to 15 mm in the third trimester. Lato et al. pointed out that if the diameter of the fetal bowel is dilated to more than 10 mm before 30 weeks of gestation, congenital intestinal atresia should be considered [13]. Similarly, in our tertiary center, bowel dilation is diagnosed when the diameter exceeds 10 mm, based on ultrasound or MRI findings. Considering that bowel diameter is related to its location and changes with gestational age and fetal development, gestational age, biparietal diameter, femoral length, and abdominal girth of the fetuses were included as reference indicators to assess the bowel dilatation more accurately as well.

The prenatal diagnosis rate of gastrointestinal malformations previously reported varies greatly in different centers, probably caused by types of gastrointestinal malformations and different examinations. C. Virgone et al. concluded that the general diagnostic rate for jejunoileal atresia detected by prenatal ultrasound in several centers was 50.6%, of which the diagnostic rate for jejunal atresia was 66.3% and that for ileal atresia was only 25.9% [1]. Patricio et al. found the combined use of ultrasound and MRI could improve the diagnostic rate of abnormal signs of the digestive tract [14]. In this study, the diagnostic accuracy of neonatal gastrointestinal malformations was 76.1% for ultrasound combined with MRI. Thereinto, the diagnostic rate for the upper gastrointestinal dilatation group was 97.6%, higher than that for the lower digestive tract dilation group, confirming that the type of gastrointestinal malformation is closely related to the bowel dilatation site. In addition, Tonni G et al. found gastrointestinal atresia in children was mostly associated with prenatal polyhydramnios [4]. John R et al. proposed the concurrence of bowel dilatation and polyhydramnios could be considered as a direction for gastrointestinal malformations such as intestinal atresia in subsequent clinical research [3]. In this research, 46 of 113 cases (40.7%) were accompanied by polyhydramnios during the fetal period, and 84.4% of them were diagnosed with gastrointestinal malformations after birth. It followed that polyhydramnios was more common in cases with gastrointestinal malformations. Therefore, taking polyhydramnios into account as well as other abnormal fetal signs like bowel dilatation site and extent can benefit a comprehensive assessment of the fetal digestive tract to recognize the risk of gastrointestinal malformation.

A double-bubble sign is a radiological finding typically seen on abdominal X-rays, ultrasound, or fetal imaging. It refers to the presence of two gas-filled loops in the upper abdomen, representing the dilated stomach and proximal duodenum. This appearance suggests obstruction in the duodenum, often due to duodenal atresia or stenosis, but can also be caused by other congenital abnormalities such as annular pancreas or intestinal malrotation. The dilated stomach and duodenum create the characteristic “double-bubble” appearance on imaging. Many studies reported the fetal double-bubble sign is associated with duodenal atresia [1,2,7,13,15], but few studies found the double-bubble sign may be related to other malformations such as annular pancreas and atresia at the beginning of the jejunum. Furthermore, the correlation between GA at the first time of diagnosis and type of malformation needs to be explored. In this study, the accuracy rate of the prenatal diagnosis for gastrointestinal malformation by double-bubble sign was 97.6%, indicating a close relationship between duodenal obstruction and double-bubble sign. In this research, the average GA at first diagnosis of bubble-sign was 26.1 ± 4.5 weeks of gestation. Only one fetus with double-bubble sign discovered at 38 + 1 weeks of gestation firstly had no gastrointestinal malformations after birth. It is suggested that the double-bubble sign caused by pathological duodenal obstruction mostly appears in the second trimester. When the double-bubble sign first appears in the last trimester, the dilation of the bowel may occur as a concomitant phenomenon, alongside the progression of gestational weeks. Meanwhile, the proportion of polyhydramnios among these cases was as high as 51.2%, suggesting that polyhydramnios can be considered as an auxiliary indicator to predict gastrointestinal obstruction.

Additionally, annular pancreas was the most common cause of duodenal obstruction in this cohort compared to duodenal atresia. We further analyzed the GA at first diagnosis of double-bubble sign between these two types of malformation. We found annular pancreas occurred more commonly in cases whose double-bubble signs were first observed in the second trimester (58.6%), while duodenal atresia occurred more often in cases diagnosed in the last trimester (54.5%). Tandler’s theory suggests that duodenal atresia is associated with the blocked vacuolization or fusion of embryonic intestinal epithelial cells and impaired lumen recanalization during weeks 9 to 12 of gestation [16]. Clinically, the most common type of duodenal atresia is membranous atresia. With small holes in the membrane, a small amount of gastrointestinal content can pass through. On the other hand, annular pancreas is usually caused by stagnation of the pancreatic bud during rotation or fusion of the dorsal and ventral sides, leading to exogenous obstruction at the descending part of the duodenum [17]. Such a process usually occurs during weeks 6 to 8 of gestation, earlier than that of duodenal atresia. It is inferred that the time of the appearance of the double-bubble sign may be related to the etiology of the duodenal obstruction. With the limitation of the small sample size in this group, how to distinguish the type of duodenal obstruction more accurately needs to be explored in more cases.

Suting Xu et al. proposed that fetal intestinal obstruction should be diagnosed according to dilatation site and GA [11]. A bowel diameter greater than 10 mm diagnosed by either fetal ultrasound or MRI met the criteria in this study. The overall diagnosis rate of gastrointestinal malformation was 63.9%, slightly higher than that reported in previous studies. Analysis of GA at first diagnosis revealed that lower digestive tract bowel dilatation occurred at 29.8 ± 4.9 weeks of gestation in cases with gastrointestinal malformation, significantly earlier than that of normal cases. Furey et al. proposed that the diameter of the normal fetal jejunoileum is approximately 2–3 mm at 22–24 weeks of gestation, and the diameter of the colon is approximately 3 mm in the second trimester [18]. In accord with the increase in GA, the diameter of the jejunoileum can increase to 5–7 mm, and the diameter of the colon can increase to 8–15 mm in the colon in the last trimester. Combined with data in this research, we should focus on the risk of intestinal malformation and use ultrasound to monitor the changes in bowel dilatation if the bowel diameter exceeds 10 mm before 30 weeks of gestation.

Manganaro et al. indicated that fetal MRI can help to detect 60.5% of abnormal signs ignored by ultrasound in fetuses with gastrointestinal malformations [2]. Particularly, fetal ultrasound is easily affected by fetal position and amniotic fluid volume. MRI can be used for the diagnosis of complex gastrointestinal abnormalities and other malformations like esophageal atresia. Patricio et al. pointed out that MRI can be used as a supplement to ultrasound since it can take an overview of the whole digestive tract to recognize intestinal obstruction [14]. The diagnosis combining these two examinations can achieve a relatively high accuracy of 84.4%. In our cohort, prenatal MRI of all neonates indicated bowel dilatation. The accuracy rate of ultrasound for gastrointestinal malformations was slightly higher than that of MRI. Tonni G et al. and Rubio EI et al. indicated that MRI can be used to observe the distribution of meconium in the fetal intestine, which helps determine the location and severity of the obstruction [4,19]. Therefore, the cases were divided into the jejunoileum group, colorectum group, and diffuse dilatation group according to the site of the dilation observed on MRI imaging. The incidence of gastrointestinal malformations in the jejunoileum group was significantly higher than that in the colorectum group. All the three cases in the diffuse dilatation group were confirmed as gastrointestinal malformations by surgery after birth. Visibly, MRI images can display the fetal abdomen comprehensively and help to find the anomaly, playing a vital role particularly in the diagnosis of intestinal atresia and stenosis. In summary, fetal ultrasound can be used to screen for gastrointestinal anomalies and to monitor progression with the advantage of high sensitivity and simplicity. MRI can be used as an auxiliary examination to make a comprehensive assessment of fetuses suspected of having gastrointestinal malformations and can play a guiding role in early postnatal treatment and surgical planning.

In our study, according to the ROC curve based on bowel diameter measures from the ultrasound, the AUC was 0.854, indicating a significant value in the prediction of gastrointestinal malformation. When the diameter of the dilated bowel exceeds 18.05 mm during the fetal period, the risk of gastrointestinal malformation increases. With a specificity of 0.968, which turned out to be the highest among all the parameters, the diameter of the bowel dilation could be used for preliminary screening in antenatal care. Previously, some scholars suggested that the diameter of the bowel will increase with gestational age, particularly in the last trimester [20]. Considering BPD, FL, and AG are widely used to evaluate fetal growth, as well as GA, we calculated the ratio of D/GA, D/BPD, D/FL, and D/AG in this research. The ROC curves were constructed correspondingly, and hence the AUC was derived. We found that the AUC for the ratio of diameter to GA, BPD, FL, and AG was 0.906, 0.873, 0.881, and 0.881, respectively. Thus, calculating the ratios of D/GA, D/BPD, D/FL, and D/AG was superior to measuring the diameter alone to predict the risk of gastrointestinal malformation. The above ratios can reflect the severity of bowel dilatation as well as its progression with fetal development more accurately. Among these, the ratio of D/GA turned out to be the best indicator for prediction with the largest AUC. The cutoff value suggested that if the ratio exceeds 0.4931, the risk of congenital gastrointestinal malformation increases significantly.

The main limitation of this study is the retrospective review of the mentioned cases. The time of prenatal examination of each case varied from the others since no fixed schedule has been proposed to monitor bowel dilation in fetuses. We recommended a monthly check of ultrasound in infants who were found with intestinal dilatation to monitor fetal growth and development and changes of intestinal dilatation.

## 5. Conclusions

The fetal double-bubble sign is closely associated with neonatal duodenal obstruction, and GA at first diagnosis is related to the etiology of the duodenal obstruction. A diameter of dilated bowel in the lower digestive tract exceeding 18.05 mm indicates the possibility of gastrointestinal malformations. When the ratio of the diameter to gestational age surpasses 0.4931, the risk of gastrointestinal malformations increases significantly.

## Figures and Tables

**Table 1:** Outcomes of children with prenatal bowel dilation.

Variables	Total (*n* = 113)
Non-malformation	27 (23.9%)
Duodenal atresia	16 (14.2%)
Annular pancreas	21 (18.6%)
Jejunal or ileal atresia	40 (35.4%)
Intestinal stricture	3 (2.7%)
Intestinal volvulus	1 (0.88%)
Abdominal cyst	3 (2.7%)
Cloacal malformation	1 (0.88%)
Meconium peritonitis	1 (0.88%)

**Table 2:** Outcomes of cases with diagnosis in different trimesters in infants with upper gastrointestinal dilatation.

	II Trimester *n* = 29 (%)	III Trimester *n* = 12 (%)	*p* Value
Polyhydramnios	14 (45.2%)	7 (58.3%)	0.431
Gastrointestinal malformation	29 (100%)	11 (91.7%)	0.293
Annular pancreas	17 (58.6%)	4 (36.4%)	0.366
Duodenal atresia	10 (34.5%)	6 (54.5%)	0.247
Jejunal atresia	2 (6.9%)	1 (9.1%)	>0.999

## Data Availability

The data that support the findings of this study are not publicly available due to ethical reasons but are available from the corresponding author upon reasonable request.
